# Dietary Supplements for Weight Management: A Narrative Review of Safety and Metabolic Health Benefits

**DOI:** 10.3390/nu14091787

**Published:** 2022-04-24

**Authors:** Eunice Mah, Oliver Chen, DeAnn J. Liska, Jeffrey B. Blumberg

**Affiliations:** 1 Biofortis Research, Addison, IL 60101, USA; 2 Friedman School of Nutrition Science and Policy, Tufts University, Boston, MA 02111, USA; nutrategic@gmail.com (O.C.); jeffrey.blumberg@tufts.edu (J.B.B.); 3 Consultant, Ridgefield, WA 98642, USA; deannliska@gmail.com

**Keywords:** cardiometabolic, diabetes, energy, body weight, BMI, liver, blood pressure

## Abstract

Dietary supplements for weight management include myriad ingredients with thermogenic, lipotropic, satiety, and other metabolic effects. Recently, the safety of this product category has been questioned. In this review, we summarize the safety evidence as well as relevant clinical findings on weight management and metabolic effects of six representative dietary supplement ingredients: caffeine, green tea extract (GTE), green coffee bean extract (GCBE), choline, glucomannan, and capsaicinoids and capsinoids. Of these, caffeine, GTE (specifically epigallocatechin gallate [EGCG]), and choline have recommended intake limits, which appear not to be exceeded when used according to manufacturers’ instructions. Serious adverse events from supplements with these ingredients are rare and typically involve unusually high intakes. As with any dietary component, the potential for gastrointestinal intolerance, as well as possible interactions with concomitant medications/supplements exist, and the health status of the consumer should be considered when consuming these components. Most of the ingredients reviewed also improved markers of metabolic health, such as glucose, lipids, and blood pressure, although the data are limited for some. In summary, weight management supplements containing caffeine, GTE, GCBE, choline, glucomannan, and capsaicinoids and capsinoids are generally safe when taken as directed and demonstrate metabolic health benefits for overweight and obese people.

## 1. Introduction

Obesity is a global epidemic that is associated with a higher risk of a multiplicity of devastating diseases, including type 2 diabetes mellitus (T2DM) and cardiovascular disease (CVD), among others [1]. The etiology of obesity is complicated, multifactorial, and involves the dysregulation of the body’s energy balance system, mediated by a complex interplay between neural, hormonal, and metabolic pathways, sedentary lifestyle, genetic predisposition, and excess calorie consumption [1,2]. Modest weight loss is considered beneficial to people with overweight/obesity due to its impact on disease risk reduction [3,4,5]. However, successful weight management, which includes not only weight loss but also weight loss maintenance (i.e., limiting weight regain), is challenging [5]. Calorie restriction and increased physical activity are the cornerstones of traditional weight management programs, but often do not lead to significant, sustained weight loss alone [6,7]. Dietary supplements, particularly those with thermogenic, lipotropic, or satiety properties, are used by many consumers to support dietary and lifestyle programs for weight management. Caffeine and green tea extract (GTE) are commonly used supplements with purported thermogenic properties. Choline and glucomannan represent supplements with expected lipotropic and satiety effects, respectively. Green coffee bean extract (GCBE) and capsaicinoids and capsinoids are newer to the market and becoming popular for weight management support. 

In the U.S., dietary supplements are regulated by the Food and Drug Administration (FDA); however, despite the existence of federal regulatory oversight, the safety of dietary supplement products targeted for weight management support has been questioned [8]. The FDA defines dietary supplements as products taken orally that contain dietary ingredient(s) intended to supplement the diet, but not intended to treat, diagnose, mitigate, prevent, or cure diseases [9]. Specifically, these products may make structure and/or function claims associated with a disclaimer stating the claims have not been reviewed by the FDA, although manufacturers are required to maintain supporting evidence [9]. Dietary supplements are also required by the FDA to be manufactured under Good Manufacturing Practices, with mandatory reporting of serious adverse events and FDA notification prior to marketing a new dietary ingredient (NDI), and manufacturers are prohibited from marketing supplements with unsafe ingredients [10]. 

Given the continued high interest in strategies for successful weight management through diet and lifestyle approaches, as well as recent questions on the safety of products in the dietary supplement weight management category, this review has identified six commonly used components that represent the breadth of the category. The safety evidence for each ingredient is reviewed, and the status of the clinical evidence on the weight management and relevant clinical health benefits are summarized. Given the large body of research for some of these ingredients, this review includes the highest quality evidence, focusing primarily on reports from authoritative sources and published systematic reviews and meta-analyses. 

## 2. Evidence Standards

Over the past several decades, much work has been directed on basing health and nutrition policy and recommendations on scientific evidence over expert opinions. Reports from authoritative bodies and government agencies that provide transparency on the scientific evidence review process are among the highest form of evidence for regulatory and policy applications. Within the biomedical sciences, systematic reviews and meta-analyses following established standards, such as PRISMA and MOOSE, and those published in peer-reviewed journals are critical resources [11,12]. The quality of the research studies included in these reviews is an important factor as well, and randomized clinical trials are the preferred evidence for establishing cause-and-effect relationships [13,14]. Other evidence, such as observational studies, can help define questions for further investigation, but because these studies are not randomized and often confounded, they may not be considered conclusive [13].

In addition, it is important that the science is assessed not only for quality but also for relevance to the key questions. For example, key aspects that authoritative bodies consider when selecting and assessing whether the evidence for decision-making is relevant generally follow the population, intervention, comparator, and outcome (PICO) approach [13,15]. In particular, the intervention tested and comparator control should be relevant and practical. Specifically, the form of delivery (supplement vs. food) and intake level (e.g., testing in the range of a typical product) are important to consider when translating science for decision-making [15]. 

For this review, authoritative sources were searched for evidence, including the U.S. FDA, the European Food Safety Authority (EFSA), Health Canada, the National Academies of Sciences and Medicine (NASEM, formerly the Institute of Medicine (IOM)), the National Institutes of Health’s Office of Dietary Supplements (ODS), and the most recent U.S. Dietary Guidelines for Americans (DGA) 2020–2025. Discussion of individual clinical trial evidence is included when more recent studies were available and/or no authoritative sources were found. In addition, to provide context around the recommended amounts of the ingredients in products provided to consumers, the Mintel Global New Products Database [16], which monitors worldwide product innovation and new product activity in consumer-packaged goods markets, with data going back to 1996, was searched for products with weight management claims (e.g., slimming, weight and muscle gain) having one or more of the ingredients of interest. 

## 3. Caffeine

Caffeine (1,3,7-trimethylxanthine) is one of the most frequently consumed dietary bioactive substances across the globe, is known as a central nervous system stimulant, and is also proposed to increase thermogenesis and fat oxidation [17,18]. Most caffeine intake is from beverage sources, including brewed coffee (56–100 mg caffeine/100 mL), instant coffee and tea (20–73 mg caffeine/100 mL), and cola soft drinks (9–19 mg caffeine/100 mL) [18]. Caffeine is generally recognized as safe (GRAS) for use in cola-type beverages by the FDA [19]. As a supplement, the ODS identifies sources of added caffeine as guarana, kola nut, yerba maté, and other herbs [17]. A search of the Mintel GNPD found 579 caffeine-containing supplements with weight management claims whereby 164 listed the caffeine content. Of these, 93% recommended intake levels of ≤400 mg/day, with the remaining 7% recommending 400 to 420 mg caffeine/day. 

Various studies on caffeine intake have been conducted, generally addressing beverage sources. In a summary of data from 18 nationally representative sources, the average caffeine consumption was found to be 23.7 mg/day, 36.6 mg/day, and 83.2 mg/day for infants, children, and adolescents, respectively, compared to 122.1–225.5 mg/day for adults [20]. These authors reported energy drinks contributed little to total caffeine intake across all groups. Specific to the U.S., examination of the 2001–2010 NHANES data showed an average daily intake of 186 mg (90th percentile, 436 mg/day) by adults from foods and beverages, including energy drinks [21]. In another study using data from the 2013–2016 NHANES, those aged 1–80 years who consume caffeine (>1 mg/d) had a mean intake of 195 mg/day, with 14% of those 30–80 years old and only 4.1% of those 13–29 years old having intakes >400 mg caffeine/day [22]. Overall, the consumption of caffeine has remained relatively stable over the past >15 years, with coffee consumers, regardless of age, being the most likely to have higher intakes. Consumption of caffeine by children is generally reported below 2.5 mg/kg/day, with the U.S. having the highest intake (average of 14 mg/day or 0.82 mg/kg/day) [20]. 

### 3.1. Caffeine Safety

The safety of caffeine has been the focus of many federal agencies and scientific and non-governmental organizations over the past several decades. In 2003, Health Canada conducted a comprehensive review concluding that an intake of ≤400 mg caffeine/day was not associated with adverse effects in healthy adults [18]. Additionally, Health Canada concluded that the consumption of ≤300 mg/day for pregnant or lactating women as well as those planning to become pregnant, and 2.5 mg/kg/day for children is not associated with adverse effects. Similarly, the EFSA has also indicated that ≤400 mg caffeine/day does not lead to safety concerns for non-pregnant adults, but identified a limit of 200 mg/day for pregnant women [23]. In 2017, an updated extensive systematic review also found consumption of ≤400 mg/day in healthy adults is not associated with overt, adverse cardiovascular, behavioral, reproductive, and developmental effects, acute effects, or bone status [24]. Additionally, the review found that the consumption of ≤300 mg caffeine/day in healthy pregnant women is generally not associated with adverse reproductive and developmental effects [24]. Limited new data were identified for children (3–12 years old) and adolescents (12–19 years old), and the authors indicated that “there is no evidence to suggest a need for a change from the recommendation of 2.5 mg/kg caffeine/day” [24]. 

The DGA 2020–2025 addressed caffeine intake and, noting that the FDA has identified ≤400 mg/day of caffeine as the amount not generally associated with dangerous, negative effects, and indicated that caffeine can be consumed ≤400 mg/day [19]. The DGA also reviewed the evidence of caffeine consumption by pregnant and lactating women. Small amounts of caffeine can pass from the mother to the infant through breast milk, but a review of the evidence indicated that consumption of ≤300 mg/day (equivalent to about 2–3 cups of coffee) by the mother does not adversely affect the infant [19]. No safe limits have been established for children aged 2 years or younger. 

When used according to the manufacturer’s instructions, most weight management supplements provide caffeine within the recommended amount of ≤400 mg caffeine/day. However, concern has been raised over the consumption of caffeine at higher intake levels. Acute intake of caffeine > 500 mg/day potentially results in various untoward events such as headache, jitteriness, agitation, anxiety, dizziness, and tinnitus [17,24]. In a review of safety data, the FDA has noted that caffeine metabolism is slowed after consumption of >500 mg, with adverse effects, such as tachycardia, ventricular arrhythmia, and seizures, at consumptions > 1200 mg [25]. Responses are variable and likely depend on individual sensitivity to caffeine, the existence of co-morbidities, and intake of concomitant medications or supplements [24]. 

Fatality due to caffeine poisoning is rare, and caffeine’s lethal dose is unclear. Reviews by Health Canada, ILSI, and FDA have concluded that there is a potential for death following acute exposures of ~10 g of caffeine for adults and adolescents [18,24,25]. However, due primarily to uncertainty in the estimates of exposure and the high risk of bias (e.g., use of case reports), there is low confidence in this evidence base. The FDA has recognized that pure or highly concentrated powdered and liquid caffeine has the potential to deliver higher amounts and thus has moved to restrict the sale of these highly concentrated items [25]. Consuming 10 g of caffeine from supplements would require ~25–26 servings of the most highly concentrated supplements, and not surprisingly, most case reports of caffeine-induced fatalities have been associated with suicide or abuse [25,26]. Very few reports of fatal caffeine poisoning in children are published [26]. 

### 3.2. Caffeine and Weight Management

Caffeine has been used as an approach in weight management because of its ability to stimulate both noradrenaline and dopamine secretions, which, in turn, may decrease BW and body fat (BF), as well as increase thermogenesis in brown adipose tissue via an unknown mechanism(s) [27]. A meta-analysis of 13 studies providing 60–4000 mg caffeine/day for 4–36 weeks showed that caffeine intake led to a reduction in BW, BF, and body mass index (BMI), and that this effect is dose-dependent [28]. However, all but three of the 13 included studies provided caffeine with other substances with potential weight loss properties. Of the three with only caffeine, one study in normal weight and one in overweight/obese subjects reported modest weight loss, whereas another in overweight/obese subjects did not. Taken together, there may be a modest effect of caffeine on weight management, but more clinical evidence with caffeine alone is needed for confirmation.

### 3.3. Caffeine and Metabolic Health 

Observational evidence suggests that caffeine may be protective against T2DM [29], [30], which has led to further investigations in this area, but results from clinical studies have been mixed. A meta-analysis of seven randomized clinical trials with primarily male normal weight participants consuming caffeine from 3–6 mg/kg [31], and a trial with healthy men and women [32] found significantly decreased insulin sensitivity indexes compared with a placebo, suggesting caffeine might possess a hyperglycemic activity by acutely impairing insulin action in adipose and muscle tissue. The hyperglycemic impact of caffeine may also extend to adolescents [33]; however, more research is needed on the long-term effect of caffeine consumption on glycemic status [29]. 

### 3.4. Caffeine Summary

Caffeine has been extensively researched and various reviews on caffeine safety, including from regulatory and authoritative sources, indicate that consumption of ≤400 mg/day for adults is safe with minimal risk of adverse events. The majority of supplements providing caffeine are within the recommended amount of ≤400 mg caffeine/day. Recommendations also indicate that pregnant and lactating women can safely consume ≤300 mg caffeine/day. Current evidence supports a beneficial effect of acute caffeine consumption on cognition, particularly on attention [34], as well as a benefit on exercise performance [35]. The evidence on the effect of caffeine on weight management is mixed, with some data suggesting caffeine might have benefits in supporting healthy blood glucose levels. Overall, as noted by the U.S. FDA “When formulated and marketed appropriately, caffeine can be an ingredient in a dietary supplement that does not present a significant or unreasonable risk of illness or injury” [25]. 

## 4. Green Tea Extract (GTE)

Green tea (*Camellia sinensis)* is one of the most popular beverages globally. Due to the association of its consumption with a wide range of health benefits, supplements formulated with GTE are of interest for a wide range of health benefits including as an antioxidant [36,37]. Extracted from green tea, GTE powder delivers polyphenols, which include flavonoids (45–90%) and caffeine (0.4–10%) at concentrations that vary for different extracts [38]. In all GTE powders, the major flavonoids are catechins, with the dominant four being epicatechin, epigallocatechin, epicatechin-3-gallate, and epigallocatechin-3-gallate (EGCG) [38]. A review of the Mintel GNPD indicates products with GTE or EGCG, when consumed under the manufacturers’ recommended servings ranged from 100–1200 mg/day and 27–350 mg/day, respectively. Some of these products also deliver 20–400 mg/day of caffeine. 

In most populations, a major source of dietary catechins is green tea infusions, which deliver an average of 0.7 mg EGCG/g of brewed green tea (~178 mg/8.5 fl oz) [39]. EFSA assessed the amount of EGCG delivered in green tea infusions and found a wide range, from 2.3 to 203 mg/100 g (~3.3 fl oz) infusion [39]. In the U.S., adults consume an average of 219 mg/day of flavonoids (~34% catechins), with tea drinkers consuming 610 mg/day flavonoids (data from NHANES 2011–2016) [40]. The EFSA panel has estimated an average EGCG intake from green tea of 90–300 mg/day, with the largest tea users consuming up to 856 mg EGCG/day [39]. EFSA also found average intakes of ECGC of 11.66–87.96 mg/day among children (3–9 years old) and 68.16–232.03 mg/day among adolescents (10–17 years old) based on food consumption surveys from various countries in Europe [39]. 

### 4.1. Green Tea Extract (GTE) Safety

The consumption of green tea infusions has traditionally been considered safe, even at levels of >5 cups/day. However, concerns have been raised regarding a link between GTE and liver function; therefore, extensive safety reviews have been conducted. In a 2017 review, Health Canada concluded that there might be a link between the use of GTE and the risk of hepatotoxicity and thus requires a cautionary statement on GTE products and limits their sale to adults [41]. EFSA published a review in 2017 and concluded that there is evidence from interventional clinical studies that intake of doses ≥800 mg EGCG/day induced a significant increase in serum transaminases (a marker of liver injury) in treated subjects compared to control [39]. 

Hu et al. conducted a comprehensive systematic review on the safety of various green tea preparations and proposed a safe intake limit of 338 mg EGCG/day for healthy adults (in a fed or fasted condition) delivered in solid dosage form based on no-observed-adverse-effect level (NOAEL) of 676 mg EGCG/day established from clinical evidence [42]. Yates et al. proposed a tolerable upper intake level (UL) of 300 mg/day of purified EGCG, based on human data in healthy adults in a fed state, and an acceptable daily intake (ADI) of 4.6 mg/kg/day, derived from animal toxicity data [43]. Dekant et al. also proposed a UL of 300 mg EGCG/day for food supplements based on clinical evidence indicating that liver effects were not observed after intakes ≤600 mg EGCG/person/day and animal toxicity studies [44]. In 2020, the USP Dietary Supplement Information Expert Committee reviewed case reports, animal data, and human clinical data and concluded the risk of hepatotoxicity due to GTE can be serious, but variable, stating that GTE rarely leads to severe hepatotoxicity in humans and its manifestation is dependent on the concentration of catechins, the bolus dose, fasting condition as well as genetic susceptibility, idiosyncrasy, and/or underlying liver health [45].

Clinical studies with beverages fortified with GTE at intakes up to 498.6 mg EGCG/person/day and durations of up to 1 year (median 12 weeks) have been published and do not show evidence of hepatotoxicity [44]. Of 15 clinical studies on GTE-containing beverages (100–704 mg EGCG/day; 1–24 weeks) reporting adverse events identified by Hu et al. [42], only five reported GI adverse events (i.e., abdominal pain and discomfort, diarrhea, and dyspepsia/indigestion) following consumption of green tea beverage and none reported liver-related adverse events. Additionally, safety recommendations for GTE are only for adults due to the limited data on children and adolescents. Limited data are available on children. In their systematic review, Hu et al. only found one study in children that assessed adverse events [42]. A clinical trial in obese Japanese children (6–16 years old; BMI > 28 kg/m^2^) consuming 576 mg catechins (30 mg EGCG)/day for 24 weeks reported no safety concerns [46]. 

### 4.2. Green Tea Extract (GTE) and Weight Management

The weight management potential of green tea has been postulated to be associated with multiple mechanisms of action, e.g., appetite control, inhibition of adipogenesis, increase in energy expenditure, and modulation of substrate oxidation [47,48,49]. The efficacy of green tea or GTE has been extensively researched, and for the most part, the results are favorable. A meta-analysis was published with 11 studies in T2DM patients, in which 10 used GTE and one used brewed green tea at amounts ranging from 400–10,000 mg/day and durations from 8–12 weeks [50]. Significant effects were reported (pooled weighted mean difference compared to control) for BW (−0.40 kg; 95% CI, −0.64 to −0.16; *p* = 0.001), BMI (−0.05; 95% CI, −0.10 to −0.00; *p* = 0.046), and BF (−0.56%; 95% CI, −0.73 to +0.38; *p* < 0.001), but not WC [50]. The results of another meta-analysis of 22 studies, which included many of the same studies of T2DM subjects, as well as additional results from studies in subjects described as overweight/obese, menopausal, and other conditions, also showed that GTE significantly decreased BW, BMI, and waist circumference (WC) [51]. Since caffeine in green tea or GTE may contribute to weight loss, a meta-analysis was conducted on 15 studies comparing green tea/GTE from 583–714 mg/day with caffeine-matched controls. The combination of green tea/GTE and caffeine led to an additional decrease in BW, BMI, and WC compared to caffeine alone over a median of 12 weeks [52]. Green tea/GTE interventions do not appear to affect obesity hormones, leptin, and adiponectin, probably due to the modest reduction in BF [53,54]. Moreover, a meta-analysis of green tea or GTE supplementation of four clinical studies with subjects having non-alcoholic fatty liver (NAFLD) reported a decrease in BMI of −2.08 kg/m^2^ (95% CI, −2.81 to −1.36) [55]. Thus, the current evidence supports the addition of GTE to lifestyle regimens for weight management.

### 4.3. Green Tea Extract (GTE) and Metabolic Health

GTE constituents can be beneficial for cardiometabolic health. The reduction in fasting blood glucose (FBG) following green tea/GTE has been supported by several meta-analyses, although the effective dose and supplementation duration are unclear [56,57,58,59,60,61]. A meta-analysis of 22 studies showed that green tea catechins with or without caffeine for >12 weeks significantly reduced FBG but did not affect insulin, HbA1c, and homeostasis model assessment-estimated insulin resistance (HOMA-IR) independent of dose [56]. Another meta-analysis of 17 studies showed that green tea/GTE significantly reduced FBG and HbA1c, especially at ≥457 mg/day, regardless of supplementation duration [57]. A more recent meta-analysis with 27 studies showed that green tea/GTE with catechin doses >500 mg/day for ≤12 weeks significantly lowered FBG [60]. However, the benefits on FBG appear not to extend to people at increased risk for diabetes or with the disease based on the results of three other meta-analysis studies [58,59,61]. These meta-analyses included subjects who were overweight/obese, normo-weight, and at risk due to other metabolic factors. Mechanisms of action of green tea catechins on glucose regulation include reducing intestinal carbohydrate digestion and absorption, inhibiting hepatic gluconeogenesis, and sensitizing insulin action [60]. 

Meta-analyses have shown that green tea/GTE reduces total cholesterol (TC) and LDL cholesterol (LDL-C) but not HDL cholesterol (HDL-C) in both normo-weight and overweight/obese subjects [62,63,64,65]. The green tea/GTE amount included in these studies ranged from 80–3000 mg catechins/day, and the reduction in TC and LDL-C appears to be independent of dose or duration. In contrast, one meta-analysis showed that GTE improved blood triglycerides (TG) but not blood cholesterol in patients with T2DM when provided at ≥800 mg/day for ≥8 weeks [66]. Additionally, the positive effect on TC, LDL-C, and TG was noted in the meta-analysis with patients with NAFLD, as well as decreases in the enzymes ALT and AST, which are indicative of liver health [55]. Obesity is a risk factor for NAFLD, and these findings suggest green tea/GTE could be an option for NAFLD patients. 

The beneficial effect of green tea/GTE on blood pressure (BP) is supported by several meta-analyses [67]. A recent meta-analysis with 24 studies providing 208–1344 mg catechins/day showed that green tea/GTE reduced systolic BP (SBP) and diastolic BP (DBP) independent of caffeine content [68]. A meta-analysis of 13 studies with diverse participant demographics, including normo-weight and overweight/obese, showed decreases in SBP and DBP following green tea catechin supplementation, whereby a catechin amount of <500 mg/day was more efficacious. Additionally, the greatest reduction in both measures was noted in participants with a baseline SBP > 130 mmHg and BMI < 30 kg/m^2^ and in studies administering GTE [69]. However, an earlier meta-analysis showed green tea/GTE remained effective in improving SBP and DBP in overweight and obese people but to a smaller extent [70]. The decrease in BP may be due to a vasodilation effect following an increase in nitric oxide. Thus, the overall evidence supports the benefits of green tea/GTE on BP in the general population and overweight/obese individuals at increased risk for CVD. 

### 4.4. Green Tea Extract (GTE) Summary

Current evidence suggests that GTE may be beneficial for weight management, glucose regulation, and reducing TC and LDL-C as well as BP in people who are overweight or obese. Undesirable effects of GTE consumption reported in clinical trials are largely GI-related tolerance issues; however, hepatotoxicity concerns have led to a proposed UL of 300 mg/day of purified EGCG. For the most part, products marketed for weight management provide EGCG at an amount at or below the proposed EGCG UL for adults when used according to the manufacturers’ instructions. 

## 5. Green Coffee Bean Extract (GCBE)

Green coffee bean extracts (GCBE) are produced from water or alcohol extraction of green coffee beans [71], and together with yerba mate (Ilex paraguariensis), are considered to be the richest source of chlorogenic acid (CGA) in nature, containing about 6–12% *w*/*w* total CGA [72]. Like other coffee products, GCBE may or may not contain caffeine. In addition, although GCBE is a rich source of CGA, roasted coffee beans, and thus coffee beverages, are not because CGA is markedly degraded during the roasting process [71,73]. CGA has diverse bioactions, including antioxidant, anti-inflammatory, glucose and lipid metabolism regulatory, and cardiovascular protective activities, which are considered the principal mechanisms underlying GCBE effects [72]. The daily intake of CGA among coffee drinkers has been estimated to be 100–300 mg/day for medium drinkers and ≤600 mg/day for heavy drinkers [74]. Per capita consumption of CGA has been estimated to be 120–594 mg/day [74]. A search of the Mintel GNPD revealed many weight management supplements containing GCBE ranging between 50–3996 mg/day. Of those that also provided CGA content, these ranged from 100–1170 mg/day. Several GCBE-containing products also include added caffeine ranging from 40–420 mg/day, whereas those that provided caffeine as a percentage of GCBE had caffeine concentrations ranging from 8–20 mg/day. 

### 5.1. Green Coffee Bean Extract (GCBE) Safety

No authoritative reports were identified specifically for GCBE; however, humans have a high metabolic capacity for CGA with no evidence of saturation of metabolic pathways for doses ≤2 g/day [75]. Animal studies suggest CGA is of low toxicity with an LD_50_ of 100 mg/kg (7 g for a 70 kg adult) [76,77]. Of the studies included in four recent meta-analyses on GCBE and cardiometabolic outcomes [78,79,80,81], only seven assessed adverse events, and of these, two reported some undesirable side effects during the GCBE or CGA intervention. One study reported stomach irritation and dizziness following 800 mg GCBE (372 mg CGA)/day for 8 weeks [81], and the other reported nausea and headache following 200 mg CGA/day for 6 weeks [82]. Both used decaffeinated supplements. Those that reported no undesirable side effects provided GCBE at 100–1000 mg/day or CGA at 54–500 mg/day for 4–24 weeks; all provided decaffeinated supplements, except for one study that provided CGA with or without 200 mg caffeine [83]. Observations from these studies suggest that CGA side effects are individual-dependent.

Considering the low occurrence of undesirable side effects reported by clinical studies testing GCBE or CGA along with the low toxicity suggested by animal studies, it is unlikely that the consumption of GCBE providing CGA ≤ 1 g (and possibly higher) would result in undesirable side effects. 

### 5.2. Green Coffee Bean Extract (GCBE) and Weight Management

Caffeine and CGA in GCBE can stimulate fatty acid oxidation and inhibit lipogenesis, which, in turn, decreases fat accumulation in the body [73]. A meta-analysis of 15 randomized clinical trials showed that GCBE in amounts ranging from 90–6000 mg/day containing 30–1200 mg/day CGA for 1–12 weeks significantly reduced BW, BMI, and WC compared to the control group, but did not affect BF and waist-to-hip ratio [84]. Additionally, the analysis did not find a dose–response relationship between CGA dosage and anthropometric measures. GCBE appears effective to support weight management but clinical studies with interventions > 12 weeks are warranted to substantiate the efficacy for weight loss and maintenance. 

### 5.3. Green Coffee Bean Extract (GCBE) and Metabolic Health

Several meta-analyses have been performed to evaluate the effect of GCBE products on cardiometabolic risk factors, such as impaired glucose regulation, dyslipidemia, and hypertension. Two meta-analyses of randomized studies showed that GCBE or CGA between 200–2000 mg/day for 2–24 weeks significantly reduced FBG and insulin, and this was not influenced by dose level [78,79]. However, the results of another meta-analysis of 10 studies found a significant decrease in FBG but not in insulin, and that the decrease in FBG was only significant for GCBE ≥400 mg/day [85]. Regarding lipid profile, a meta-analysis of 13 randomized studies showed that GCBE or CGA between 200 and 2000 mg/day for 2–24 weeks significantly reduced fasting blood TC but not TG, LDL-C, or HDL-C [78]. Finally, the beneficial effect of GCBE on SBP and DBP was demonstrated in a meta-analysis of nine studies in people with hypertension, whereby the effect size in SBP was larger with GCBE doses ≥400 mg/day [86]. In that meta-analysis, the hypertensive subjects were diverse and included overweight/obese and those with metabolic syndrome. These results appear most supportive of GCBE in blood glucose while the effect of GCBE on BP and lipids is promising but requires further investigation.

### 5.4. Green Coffee Bean Extract (GCBE) Summary

Considering the low occurrence of undesirable side effects reported in clinical studies providing GCBE or CGA along with the low toxicity suggested by animal studies, it is unlikely that consumption of GCBE or CGA through weight management supplements is harmful. Limited clinical evidence supports a potential beneficial effect of GCBE for short-term (<12 weeks) weight loss and management of blood glucose and BP but not that of lipids [87], and CGA exhibits anti-diabetic and anti-lipidemic properties [73].

## 6. Choline

Choline is an essential water-soluble micronutrient that exerts diverse functions in cellular maintenance and growth throughout one’s lifetime. Specifically, choline is involved in metabolic pathways for the synthesis of acetylcholine, betaine, phospholipids, and trimethylamine, which, in turn, play roles in lipid transport, membrane synthesis, neurotransmission, and one-carbon metabolism [88,89]. Choline is particularly important for fetal brain development, and low choline status or deficiency is associated with negative health outcomes. The NASEM Dietary Reference Intake Report on choline has defined adequate intakes (AI) for the U.S. population that range between 425–550 mg/day for adults, 375–550 mg/day for adolescents aged 14–18 years, 250 mg/day for children age 4–8 years, 200 mg/day for young children (1–3 years old), and 125–150 mg/day for infants up to 1 year old [90]. Similarly, the EFSA set the recommended AI for choline at 400–550 mg/day for all adults and adolescents 14 years old and above, 375 for adolescents 9–13 years old, and 150–250 mg/day for infants and young children [91].

Average dietary choline intake in several countries has been reported to be below recommendations for most populations and age/sex groups, ranging from 230–468 mg/day for adults, and mostly from animal-based food sources [89]. For example, in the U.S., the average choline intake of men and women is 421 mg/day and 279 mg/day, respectively [88]. The DGA 2020–2025 has addressed choline nutriture and noted that choline intake needs to be higher during pregnancy and lactation for replenishment of maternal stores and support for fetal development, particularly of the brain and spinal cord [19]. Given that most women do not meet recommended intakes of choline during pregnancy and lactation, particularly women following a vegetarian or vegan dietary pattern, and that most prenatal vitamins do not contain choline, the DGA also states that supplementation may be necessary [19]. 

In dietary supplements, choline is delivered in different forms, including phosphatidylcholine, CDP-choline (citicoline), L-alpha-glycerylphosphorylcholine (alpha-GPC), and choline salts (e.g., choline bitartrate, choline chloride). A search of the Mintel GNPD database revealed some supplements with weight management claims that contain choline, whereby a majority were in the form of choline bitartrate, with a handful as citrate salts, phosphatidylcholine, and alpha-GPC. Most of these products appear to include choline as a vitamin for nutrition purposes, but some claim that the added choline helps with fat metabolism and increases energy. Among the products that listed the amount of choline per serving, most provide <300 mg/day, with the highest providing <993 mg/day. Meanwhile, most top-selling choline supplements are marketed for cognition and provide between 200–500 mg choline/day, with the highest being 1000 mg/day [92]. Thus, compared to other types of choline supplements, those marketed for weight management are not more likely to substantially contribute to excess dietary choline. 

### 6.1. Choline Safety

The 1998 Dietary Reference Intake report established upper levels of choline based on their review of safety data and the identification of the LOAEL as 3.5 g/day in adults, 3 g/day for adolescents 14–18 years old, 2 g/day for children aged 9–13 years, and 1 g/day for children aged 1–8 years [90]. The daily oral administration of high choline levels, such as 10 g choline chloride (7.5 g choline), has produced a slight hypotensive effect in patients with Alzheimer’s disease, and mild reports of transient Parkinsonian signs (bradykinesia, tremor, and rigidity) were observed at 12.7 g/day of choline chloride in people with tardive dyskinesia [90]. Excess choline intake (e.g., 10–16 g/day choline) has also been associated with fishy body odor, vomiting, salivation, sweating, and GI effects in patients with tardive dyskinesia and cerebellar ataxia, as well as generation of the metabolite trimethylamine-N-oxide [90,91]. However, as noted by EFSA, “these are indirect adverse effects of choline, depending on a ‘high’ dietary amount and a specific gut microbiome” [91]. Given that choline is a required micronutrient, and most people consume lower than the recommended amount, a main focus in the literature has been on inadequate intakes, not excess consumption. 

### 6.2. Choline and Weight Management

Clinical evidence on choline and weight loss is scarce, with no authoritative reports or systematic reviews specifically on choline and weight management identified. Based on its known lipotropic activity, some clinical studies have explored its effect on fat deposition and weight loss. In clinical studies, one report with 22 young female athletes undergoing normal athletic training found 1-week supplementation of 2 g choline/day led to a larger rapid weight loss as compared to the placebo [93]. Reduced BF accounted for the majority of the weight loss, mainly due to the effect of choline on fat metabolism. However, another study testing the effect of 6-week supplementation of 500 or 2000 mg/day citicoline (CDP-choline, a dietetic source of choline) in 16 overweight adults did not affect BW despite a lower appetite rating at the high dose compared to the baseline [94]. An observational study of 3054 adults in Newfoundland, Canada showed that dietary choline intake was associated inversely with BW, BMI, WC, BF, and positively with lean mass in both women and men [95], but an association was not found in an observational study with 788 6-year-old Iranian children [96].

### 6.3. Choline and Metabolic Health

Some evidence is available for the impact of choline on obesity-related conditions. For example, choline plays a key role in fat metabolism in the liver via phosphatidylcholine, a product of choline metabolism, which is essential for the assembly/secretion of very low-density lipoproteins [89,97]. Mechanisms including abnormal phospholipid synthesis, defects in lipoprotein secretion, and oxidative damage can exacerbate liver damage and contribute to liver disorders such as NAFLD. A link between choline deficiency and hepatic lipid disposition has been recognized for over 50 years [98]. The significance of choline in NAFLD is manifested in the occurrence of hepatic steatosis with choline deficiency and the reversal of lipid infiltration with choline replacement in patients receiving total parenteral nutrition [99]. Additionally, decreased choline intake was found to be significantly associated with an increased incidence of hepatic fibrosis in postmenopausal women with NAFLD [100]. Moreover, higher dietary choline intake was associated with a 28% lower risk of NAFLD only in normal-weight Chinese women but not in overweight/obese women or men [101]. Although clinical evidence on the efficacy of choline supplementation in protecting against the NAFLD development and progression is lacking [102,103], the current evidence suggests the potential of choline for nutritional support and management of NAFLD.

Choline serves as a dietary precursor for the gut microbial-generated trimethylamine (TMA), which is oxidized by the hepatic enzyme flavin-containing monooxygenase 3 (FMO3) to form trimethylamine-N-oxide (TMAO) [104,105]. High levels of TMAO have been linked to detrimental vascular and inflammatory effects in animal and cell culture studies. In humans, increased levels of TMAO have been associated with greater CVD risk, although it remains unclear whether this relationship is a non-pathogenic indirect marker, or if TMAO has a direct role in disease progression [104]. Further, many of the choline-rich foods that may contribute to TMAO are considered cardioprotective, and the contribution of their consumption to clinically relevant levels of TMAO is unknown [104,105]. Therefore, the role of TMAO, and choline as a precursor, remains unclear. Choline supplementation has been explored in randomized, double-blind, placebo-controlled trials on lipid profiles with no effect seen with 400 mg/day choline in T2DM subjects [106] or with 1 g choline/day in 42 healthy postmenopausal women [107]. The supplementation of betaine, which is a metabolite of choline produced in the human body, at 1.5, 3, and 6 g/day for 6 weeks, significantly increased TC, LDL-C, and TG compared to placebo [108]. In the same study, 2-week supplementation with 2.6 g/day of choline (provided as phosphatidylcholine) increased serum TG by (8%) but did not alter TC, LDL-C, or HDL-C. Taken together, although choline is involved in fat metabolism, its effect following supplementation on lipid profile in humans is equivocal. 

### 6.4. Choline Summary

Choline is an essential micronutrient with lipotropic activity that helps catalyze fat metabolism and prevent fat disposition in the liver. The clinical evidence on the efficacy of choline supplementation on weight management is limited and inconsistent. Given that choline is a required micronutrient, and most people consume less than the recommended intake, the main focus in the literature has been on inadequate intakes, not excess consumption. 

## 7. Glucomannan

Glucomannan is a high molecular-weight polysaccharide mainly composed of d-mannose and d-glucose linked by β-1,4 glycosidic bonds with side chains [109]. Although it can be present in other plants, such as lily and orchid, glucomannan for human consumption is commonly derived from the tuber or root of *Amorphophallus konjac* or elephant yam [110]. Glucomannan is a non-digestible carbohydrate that has been approved for labeling as a dietary fiber by the FDA based on its ability to attenuate cholesterol levels [111]. As a fiber, glucomannan is not digested in the upper GI and thus is available for fermentation by gut microbiota. 

Like other fibers, glucomannan has been promoted for effects on weight management and associated benefits, such as glucose and cholesterol-lowering, laxative, prebiotic, and anti-inflammatory activities. It is a hygroscopic fiber, however, which puts it in a subcategory of fibers, such as guar gum, which are viscous and able to form a large volume of mucilage after absorbing water in the upper GI tract. The mucilage can then affect satiety as well as nutrient digestion, absorption, and metabolism in the GI tract [109]. 

Overall, dietary fiber has been identified as a nutrient of public health concern in all age/sex groups of the U.S. population, with more than 90% of females and 97% of males not meeting recommended levels of 21–38 g/day for most adults and 19–38 g/day for children aged 1–18 years [19,112]. A search of the Mintel GNPD indicated the recommended intake of glucomannan for weight management products containing glucomannan ranged from 10–3330 mg/day.

### 7.1. Glucomannan Safety 

Following their review of animal and human studies of konjac food supplements, the EFSA concluded there was no safety concern at <3 g/day konjac intake for the general population and noted that abdominal discomfort, including diarrhea or constipation, may occur after this daily dose in adults [113]. This advice is similar to that for other fibers, which can cause untoward GI side effects, particularly if large amounts are consumed without a stepped-up dosing regimen to allow for acclimation. Usually, these GI symptoms, if they occur, are mild to moderate and transient and not a safety issue. For example, a meta-analysis of randomized weight loss clinical trials in adults found GI-related symptoms in six of eight studies that assessed adverse events when konjac glucomannan was provided at 1.5–10 g for 3–12 weeks [114]. Another meta-analysis of randomized studies on the effect of 3 g for 12 weeks and 3.99 g for 8 weeks konjac glucomannan supplementation on BW also reported GI-related symptoms in two of the six studies that assessed adverse events [115]. Similarly, GI-related adverse events were also reported by some studies in children; however, in two studies in obese children, no adverse events were reported with 2–3 g/day glucomannan in capsules [116,117]. Another study in children (6–17 years old) provided 3 g/day noted that both the active and placebo (maltodextrin) groups experienced a similar number of adverse events that were mostly GI related [118]. 

Glucomannan aids in weight loss due to its hygroscopic property, whereby it swells rapidly in the stomach to produce a feeling of satiety and fullness. Water-soluble gums, such as konjac glucomannan, psyllium, and guar gum, may cause esophageal obstructions if the dry powder is consumed with inadequate water, thus allowing expansion to occur in the esophagus. The risk of esophageal obstructions for glucomannan products is dependent on the amount of glucomannan and how the products are manufactured, as well as individual consumer characteristics (e.g., ability to swallow, esophageal anatomy, compliance to consumption instructions, etc.) [119]. The FDA has reviewed this category of ingredient related to safety and requires that over-the-counter products in a dry or incompletely hydrated form must carry a warning indicating the product should be taken with adequate water and should not be consumed by individuals who have difficulty swallowing [120]. Specific to dietary supplements, the ODS has stated, “Significant safety concerns reported for tablet forms, which might cause esophageal obstructions, but few safety concerns with up to 15.1 g/day of other forms for several weeks” [17]. 

### 7.2. Glucomannan and Weight Management

In 2010, the EFSA approved a reduction for BW claim for glucomannan at a daily dose of ≥3 g consumed over three eating occasions throughout the day and in the context of an energy-restricted diet in overweight adults [121]. However, two meta-analyses published after the EFSA approval reported inconsistent results. A 2014 meta-analysis of eight human studies providing 1–3 g glucomannan/day reported a non-significant reduction of 0.5 kg [114], and a 2021 meta-analysis of eight studies reported a 1.27 kg weight reduction [122]. Thus, the data to date are inconsistent. The role of the microbiota in healthy weight management is of high interest and in vitro studies have shown konjac fiber may beneficially alter the human gut microbiome [123]; however, human data on overweight/obese subjects are scarce. In a randomized, double-blind, controlled study, konjac flour, which provides primarily konjac glucomannan, resulted in a reduction in BMI and fat mass and significantly increased α-diversity of the gut microbiome in 69 obese subjects over 5 weeks [124].

Maintaining lean muscle mass is an important issue in weight management regimes. Supplementation of 3 g/day of konjac glucomannan with 300 mg calcium carbonate for 60 days decreased BW by 1.2 kg and BF by 1.1 kg while maintaining lean muscle mass in compliant overweight participants compared to the placebo (300 mg of calcium carbonate alone) [125]. More studies on glucomannan in weight management are needed to confirm these findings and determine optimum clinical application conditions. In addition, understanding the impact of changes in physiochemical properties of glucomannan (e.g., high vs. low polymerization) is also an important area for future research. 

### 7.3. Glucomannan and Metabolic Health

Glucomannan has been investigated for its effects on many obesity-related conditions [126]. The FDA has reviewed data on konjac glucomannan and its effect on blood lipids for determining a beneficial physiological effect and thus its ability to be labeled as a fiber [111]. Their review indicated nine studies from which conclusions could be drawn had been published on this effect, with six of these studies showing a reduction in TC and/or LDL-C [111]. The evidence included children and adults with hypercholesterolemia, with amounts of glucomannan between 2–13 g/day delivered in a capsule or a food product, over 3–8 weeks [111]. Due to the delay in gastric emptying and interference of nutrient accessibility for digestion and absorption, glucomannan is anticipated to also impact blood glucose [110]. The results of another meta-analysis with glucomannan amounts ranging from 1.2–15.1 g/day showed significant decreases in FBG, TC, LDL-C, non-HDL-C, and TG in a diverse population including obese, hyperlipidemic, and diabetic adults [127]. Subgroup analysis did not show similar results in pediatric subjects. A more recent meta-analysis of 12 studies similarly reported that Konjac glucomannan significantly lowered LDL-C, and non-HDL-C, but not apolipoprotein B [128]. Recent clinical studies have also suggested that glucomannan may influence carbohydrate digestion and absorption when it is co-consumed with high-carbohydrate foods [129,130]. 

### 7.4. Glucomannan Summary 

Glucomannan has an EFSA-approved claim for weight loss; however, the effect on weight loss is modest and more investigations are warranted to confirm the consistency of this effect. Existing clinical evidence supports beneficial effects on blood glucose, lipid profile, and GI function. Like other fibers, consumption of glucomannan is expected to cause transient undesirable GI side effects in some people, particularly if a large amount is consumed without allowing for acclimation to the increase in fiber intake. Consumers of glucomannan supplements are urged to follow the manufacturer’s consumption instructions, consume adequate liquid with glucomannan products, and be aware of any deficits in swallowing or esophageal anatomy that could increase their risk for esophageal obstruction. 

## 8. Capsaicinoids and Capsinoids

Capsaicinoids are the active ingredients that give chili peppers their characteristic pungent flavor [131]. Capsaicinoids are a family of alkaloids that include capsaicin, dihydrocapsaicin, nordihydrocapsaicin, homocapsaicin, and homodihydrocapsaicin, with capsaicin being the dominant capsaicinoid. Capsaicin has been traditionally used for muscular pain, headaches, and GI protection and to improve circulation [132]. Understanding the actions of capsaicin led to the discovery of its binding intracellularly to Transient Receptor Potential Vanilloid subfamily member 1 (TRPV1) that is expressed predominantly in sensory neurons, and whose activation can regulate pain sensation [133]. More recently, capsaicinoids have been investigated for impact on several lifestyle health outcomes, including weight management and obesity-related conditions [131]. A similar but independent group of compounds named capsinoids has also been investigated in several clinical trials on weight management [134]. Unlike capsaicinoids, capsinoids are non-spicy compounds and include capsiate (4-hydroxy-3-methoxybenzyl [E]-8-methyl-6-nonenoate), dihydrocapsiate, and nordihydrocapsiate.

The consumption of capsaicinoids across different countries ranges from 1–30 mg/day. Average consumption in Korea is ~3.25 mg/day, and consumption in Mexico averages 24.5–32 mg/day, though intake up to 250 mg/day has been reported [135,136]. A search of the Mintel GNPD database indicated weight management supplements recommend <10 mg capsaicin/day or <10 mg dihydrocapsiate/serving.

### 8.1. Capsaicinoids and Capsinoids Safety

Few authoritative and government reports related to capsaicinoids safety have been published. The ODS has stated that few safety concerns have been reported for ≤33 mg/day for 4 weeks or 4 mg/day for 12 weeks of capsaicin and other capsaicinoids [17]. Capsaicinoids have been evaluated for food uses as well. The capsinoid dihydrocapsiate is GRAS at 1 and 3 mg/serving, and this conclusion has been submitted to the FDA with no objection from the agency [137]. EFSA has also concluded that dihydrocapsiate is safe for use as a food ingredient or food uses [137]. A NDI 75-day premarket notification for dihydrocapsiate marketed as a dietary ingredient (i.e., supplement) with a maximum daily intake of 15 mg is on file with the FDA [138]. Related to supplements, EFSA evaluated phenylcapsaicin, a chemically synthesized analog of capsaicin for use in food supplements and foods for special medical purposes at a maximum of 2.5 mg/day in the general population above 11 years old [139]. In contrast, a meta-analysis [140] on the thermogenic properties of capsaicinoids reported that intervention amounts of capsaicin supplements were generally low at 0.2–7 mg, with one study providing a single acute intervention of 150 mg/day [141,142] and another providing 135 mg/day for 3 months [143]. The study providing 150 mg/day [141,142] did not assess adverse effects, whereas the study providing 135 mg/day [143] noted that 10 of the 42 participants who were randomized to the capsaicin group “complained” about the capsules, but no further details on the complaints were provided. 

Some safety data are available from clinical studies; however, many meta-analyses combine data of foods that contain capsaicinoids with that of supplement delivery of the concentrates or isolated components. For example, a meta-analysis of metabolic syndrome clinical studies providing capsaicinoids ranging from 2–9 mg/day for 4–12 weeks combined food and supplement sources noted no serious adverse events and no events leading to withdrawal were reported in the studies [144]. Ten of the 12 studies included in this analysis used a supplement form ranging from fermented red pepper paste pills, red pepper capsules, capsinoid capsules, and dihydrocapsiate capsules. Eight of the 10 studies using supplements reported adverse events, with six indicating no adverse events during the study [144]. The other two studies indicated some tolerance-related adverse events of leg cramps (with 3 or 9 mg/day dihydrocapsiate over 4 weeks), dyspepsia, bowel irregularities, and skin rash (with 9 mg capsinoids capsules over 12 weeks) [144]. A tolerability study of a proprietary product comprising of a blend of capsaicinoids obtained from *Capsicum annuum* has been reported as well and showed no change in tolerability at ≤500 mg/day (10 mg/day of capsaicinoids) when provided for 1 week [145]. 

A controversial area of safety has been the proposed carcinogenic properties of hot peppers. Early studies assessed the food and not capsaicinoid or capsinoid extracts but attributed putative effects to capsaicin. This has complicated the available data. For example, a 2014 meta-analysis reported that low intake of capsaicin showed protection, whereas medium-high intakes showed susceptibility to gastric cancer, but this only searched for studies that assessed chili pepper food and only included case–control design studies [146]. Similarly, more recent meta-analyses have shown inconsistent relationships but have evaluated the whole food and not supplements [147,148]. Interestingly, a nonlinear association of gastric cancer risk with capsaicin intake was shown with potential protective effects at intakes of chili delivering 0–30 mg capsaicin/day, no clear relationship at 30–90 mg/day, and risk at >90 mg/day [148]. These all represented observational studies, and therefore, no causal relationship can be established from these data. Moreover, supplement compositions are variable and most intervention studies on health effects are conducted at amounts much lower than those associated as a putative risk factor for gastric cancer. Information on gastric cancer risk needs to be determined for the supplement forms of these ingredients, and more data are needed to understand if a protective effect exists at lower levels. 

### 8.2. Capsaicinoids and Capsinoids and Weight Management

The consumption of capsaicinoid-containing foods has been associated with a lower incidence of obesity, leading to an interest in the role of capsaicinoids themselves [149]. The EFSA evaluated health claims for capsaicin and maintenance of weight loss and increased carbohydrate oxidation in 2011 and found limited evidence [150]; however, since then, several new studies have been published. A meta-analysis reported a marginal decrease in BW and BMI when assessing results from four and five studies, respectively [144]. 

The effect of capsaicinoids has also been explored for helping promote a negative energy balance through increased energy expenditure (EE) and increasing fat oxidation [140]. For example, a meta-analysis of nine studies with different durations and amounts showed that capsaicinoids significantly increased fat oxidation and EE (+58.56 kcal/day) [151]. Additionally, Ludy et al. [140] conducted a meta-analysis to examine whether there was a dose-dependent effect of capsaicin on EE and fat oxidation, and found that a positive effect on EE was only noted for red pepper or capsaicin delivering 135–150 mg/day but not for amounts < 35 mg. This increased EE can be a consequence of enhanced brown adipose density and activity, which is involved in thermogenesis [152,153]. Capsaicinoids may also affect food intake, and a meta-analysis of eight studies showed that capsaicinoid ingestion with a minimum dose of 2 mg before a meal significantly decreased ad libitum calorie (−74.0 kcal) during the meal, suggesting an appetite suppression effect [154]. A novel study with an intraduodenal capsaicin infusion of 1.5 mg reported significantly increased satiety, likely related to GI stress but not to satiety hormones, such as PYY or GLP-1 [155]. Nevertheless, these observations are not supported by the results of another meta-analysis that found a non-significant reduction in BW of −0.19 kg [144], although a limitation of this study is the heterogeneity of the interventions, which include capsaicin supplements and hot pepper foods. 

### 8.3. Capsaicinoids and Capsinoids and Metabolic Health

Preclinical evidence shows that capsaicinoids can modulate glucose homeostasis and lipid metabolism through the TRPV1-dependent pathway and others [156]. Clinical trials have been conducted on FBG and have shown a null effect [157,158]. However, these null results could be ascribed to study participants having normal glucose regulation. No significant changes in lipid profile were reported in overweight adults after 12 weeks of capsaicinoid supplementation at 2 or 4 mg/day [157]. However, HDL-C increased and TG decreased following 4 mg capsaicin/day for 3 months in adults with low HDL-C [157]. Null results were reported for SBP and DBP in two meta-analyses of studies in normotensive participants [159,160]. Taken together, the evidence of capsaicinoids and capsaicinoid-containing foods on glucose regulation and lipid profile is equivocal, so more studies are warranted, particularly in populations with overweight and obesity. 

### 8.4. Capsaicinoid and Capsainoid Summary

Capsaicinoids have been shown to increase EE, although subsequent effects on weight loss are modest. Similarly, the evidence of capsaicinoids and capsainoids on glucose regulation and lipid profile is inconsistent and limited. Weight-loss supplements typically provide <10 mg capsaicin/day. Although safety data are confounded by data on high levels of acute hot pepper foods in some, few safety concerns have been reported in clinical trials with capsaicinoid/capsinoid supplements at ≤33 mg/day for 4 weeks and lower amounts for longer periods. Based on these observations, the ODS has stated few safety concerns have been reported for 33 mg/day for 4 weeks or 12 mg/day for 12 weeks for capsaicin and other capsaicinoids. 

## 9. Conclusions

Eighty percent of Americans take dietary supplements as part of a healthy lifestyle, including weight management products [161]. We reviewed six ingredients that are representative of the broad range of dietary supplements used for weight management—caffeine, GTE, GCBE, choline, glucomannan, and capsaicinoids and capsainoids—for safety as well as evidence related to weight management and metabolic health. Published authoritative reports and comprehensive systematic reviews were available for assessing the safety of caffeine, GTE, choline, and glucomannan, with information on the main bioactive of GCBE extract (i.e., CGA) also well studied. Further, caffeine, GTE (specifically EGCG), and choline have recommended intake limits. Most dietary supplement products marketed for weight management that include these ingredients have levels within the amounts identified as generally safe in these reports. Serious events are rare and often involve intentionally high intakes; however, as with any dietary component, these ingredients have the potential for intolerance effects, such as GI symptoms, and inter-individual variations, concomitant medications/supplements, and health status may also play a role in these sensitivities. 

Calorie restriction, improvements in dietary quality, and physical activity are key components in weight management programs, and successful approaches are multifactorial. Due to this complexity, weight management studies that assess dietary supplements in the context of other modalities can lead to challenges in discerning the effect of a specific dietary ingredient. In our review of authoritative reports and human clinical research, most of the ingredients appear to improve some aspects of metabolic health in people with overweight and obesity, such as supporting healthy blood glucose, lipids, and BP. Of these components, GTE has the most plentiful and consistent clinical evidence for weight management benefits. In summary, supplements providing caffeine, GTE, GCBE, choline, glucomannan, and/or capsaicinoids and capsainoids may provide some benefit for weight management and related measures of metabolic health and are generally safe when used in accordance with the manufacturers’ directions. 

## Data Availability

Not applicable.

## References

[1] Larsson S.C., Burgess S. (2021). Causal role of high body mass index in multiple chronic diseases: A systematic review and meta-analysis of Mendelian randomization studies. BMC Med..

[2] Kumar R.B., Srivastava G., Reid T.J., Aronne L.J. (2021). Understanding the pathophysiologic pathways that underlie obesity and options for treatment. Expert Rev. Endocrinol. Metab..

[3] Klein S., Burke L.E., Bray G.A., Blair S., Allison D.B., Pi-Sunyer X., Hong Y., Eckel R.H. (2004). Clinical Implications of Obesity With Specific Focus on Cardiovascular Disease. Circulation.

[4] Williamson D.A., Bray G.A., Ryan D.H. (2015). Is 5% weight loss a satisfactory criterion to define clinically significant weight loss?. Obes. Silver Spring.

[5] Heymsfield S.B., Aronne L.J., Eneli I., Kumar R.B., Michalsky M., Walker E., Wolfe B.M., Woolford S.J., Yanovski S. (2018). Clinical Perspectives on Obesity Treatment: Challenges, Gaps, and Promising Opportunities. NAM Perspectives. Discussion Paper. https://nam.edu/.

[6] Tobias D.K., Hall K.D. (2021). Eliminate or reformulate ultra-processed foods? Biological mechanisms matter. Cell Metab..

[7] Ludwig D.S., Aronne L.J., Astrup A., de Cabo R., Cantley L.C., Friedman M.I., Heymsfield S.B., Johnson J.D., King J.C., Krauss R.M. (2021). The carbohydrate-insulin model: A physiological perspective on the obesity pandemic. Am. J. Clin. Nutr..

[8] Geller A.I., Shehab N., Weidle N.J., Lovegrove M.C., Wolpert B.J., Timbo B.B., Mozersky R.P., Budnitz D.S. (2015). Emergency Department Visits for Adverse Events Related to Dietary Supplements. N. Engl. J. Med..

[9] FDA Questions and Answers on Dietary Supplements. US Food and Drug Administration. https://www.fda.gov/food/information-consumers-using-dietary-supplements/questions-and-answers-dietary-supplements.

[10] Dwyer J.T., Coates P.M., Smith M.J. (2018). Dietary Supplements: Regulatory Challenges and Research Resources. Nutrients.

[11] Page M.J., Moher D., Bossuyt P.M., Boutron I., Hoffmann T.C., Mulrow C.D., Shamseer L., Tetzlaff J.M., Akl E.A., Brennan S.E. (2021). PRISMA 2020 explanation and elaboration: Updated guidance and exemplars for reporting systematic reviews. BMJ.

[12] Stroup D.F., Berlin J.A., Morton S.C., Olkin I., Williamson G.D., Rennie D., Moher D., Becker B.J., Sipe T.A., Thacker S.B. (2000). Meta-analysis of observational studies in epidemiology: A proposal for reporting. Meta-analysis Of Observational Studies in Epidemiology (MOOSE) group. JAMA.

[13] FDA Guidance for Industry: Evidence-Based Review System for the Scientific Evaluation of Health Claims. https://www.fda.gov/regulatory-information/search-fda-guidance-documents/guidance-industry-evidence-based-review-system-scientific-evaluation-health-claims.

[14] Blumberg J., Heaney R.P., Huncharek M., Scholl T., Stampfer M., Vieth R., Weaver C.M., Zeisel S.H. (2010). Evidence-based criteria in the nutritional context. Nutr. Rev..

[15] Dwyer J.T., Rubin K.H., Fritsche K.L., Psota T.L., Liska D.J., Harris W.S., Montain S.J., Lyle B.J. (2016). Creating the Future of Evidence-Based Nutrition Recommendations: Case Studies from Lipid Research. Adv. Nutr..

[16] Mintel Global New Products Database (GNPD). https://www.mintel.com/global-new-products-database.

[17] NIH Office of Dietary Supplements (2021). Dietary Supplements for Weight Loss. Fact Sheet for Health Professionals.

[18] Nawrot P., Jordan S., Eastwood J., Rotstein J., Hugenholtz A., Feeley M. (2003). Effects of caffeine on human health. Food Addit. Contam..

[19] U.S. Department of Agriculture and U.S. Department of Health and Human Services (2020). Dietary Guidelines for Americans, 2020–2025.

[20] Verster J.C., Koenig J. (2018). Caffeine intake and its sources: A review of national representative studies. Crit. Rev. Food Sci. Nutr..

[21] Fulgoni V.L., Keast D.R., Lieberman H.R. (2015). Trends in intake and sources of caffeine in the diets of US adults: 2001–2010. Am. J. Clin. Nutr..

[22] Benson S.M., Unice K.M., Glynn M.E. (2019). Hourly and daily intake patterns among U.S. caffeinated beverage consumers based on the National Health and Nutrition Examination Survey (NHANES, 2013–2016). Food Chem. Toxicol..

[23] EFSA NDA (EFSA Panel on Dietetic Products, Nutrition and Allergies) (2015). Scientific Opinion on the Safety of Caffeine. EFSA J..

[24] Wikoff D., Welsh B.T., Henderson R., Brorby G.P., Britt J., Myers E., Goldberger J., Lieberman H.R., O’Brien C., Peck J. (2017). Systematic review of the potential adverse effects of caffeine consumption in healthy adults, pregnant women, adolescents, and children. Food Chem. Toxicol..

[25] FDA Guidance for Industry: Highly Concentrated Caffeine in Dietary Supplements. https://www.fda.gov/regulatory-information/search-fda-guidance-documents/guidance-industry-highly-concentrated-caffeine-dietary-supplements.

[26] Cappelletti S., Piacentino D., Fineschi V., Frati P., Cipolloni L., Aromatario M. (2018). Caffeine-Related Deaths: Manner of Deaths and Categories at Risk. Nutrients.

[27] Van Schaik L., Kettle C., Green R., Irving H.R., Rathner J.A. (2021). Effects of Caffeine on Brown Adipose Tissue Thermogenesis and Metabolic Homeostasis: A Review. Front. Neurosci..

[28] Tabrizi R., Saneei P., Lankarani K.B., Akbari M., Kolahdooz F., Esmaillzadeh A., Nadi-Ravandi S., Mazoochi M., Asemi Z. (2019). The effects of caffeine intake on weight loss: A systematic review and dos-response meta-analysis of randomized controlled trials. Crit. Rev. Food Sci. Nutr..

[29] Neves J.S., Leitão L., Magriço R., Bigotte Vieira M., Viegas Dias C., Oliveira A., Carvalho D., Claggett B. (2018). Caffeine Consumption and Mortality in Diabetes: An Analysis of NHANES 1999–2010. Front. Endocrinol. Lausanne.

[30] Grosso G., Godos J., Galvano F., Giovannucci E.L. (2017). Coffee, Caffeine, and Health Outcomes: An Umbrella Review. Annu. Rev. Nutr..

[31] Shi X., Xue W., Liang S., Zhao J., Zhang X. (2016). Acute caffeine ingestion reduces insulin sensitivity in healthy subjects: A systematic review and meta-analysis. Nutr. J..

[32] Beaudoin M.S., Allen B., Mazzetti G., Sullivan P.J., Graham T.E. (2013). Caffeine ingestion impairs insulin sensitivity in a dose-dependent manner in both men and women. Appl. Physiol. Nutr. Metab..

[33] Shearer J., Reimer R.A., Hittel D.S., Gault M.A., Vogel H.J., Klein M.S. (2020). Caffeine-Containing Energy Shots Cause Acute Impaired Glucoregulation in Adolescents. Nutrients.

[34] Irwin C., Khalesi S., Desbrow B., McCartney D. (2020). Effects of acute caffeine consumption following sleep loss on cognitive, physical, occupational and driving performance: A systematic review and meta-analysis. Neurosci. Biobehav. Rev..

[35] Grgic J., Grgic I., Pickering C., Schoenfeld B.J., Bishop D.J., Pedisic Z. (2020). Wake up and smell the coffee: Caffeine supplementation and exercise performance-an umbrella review of 21 published meta-analyses. Br. J. Sports Med..

[36] Pastore R.L., Fratellone P. (2006). Potential health benefits of green tea (Camellia sinensis): A narrative review. Explore.

[37] Rasaei N., Asbaghi O., Samadi M., Setayesh L., Bagheri R., Gholami F., Soveid N., Casazza K., Wong A., Suzuki K. (2021). Effect of Green Tea Supplementation on Antioxidant Status in Adults: A Systematic Review and Meta-Analysis of Randomized Clinical Trials. Antioxidants.

[38] Chacko S.M., Thambi P.T., Kuttan R., Nishigaki I. (2010). Beneficial effects of green tea: A literature review. Chin. Med..

[39] Younes M., Aggett P., Aguilar F., Crebelli R., Dusemund B., Filipic M., Frutos M.J., Galtier P., Gott D., EFSA Panel on Food Additives Nutrient Sources added to Food (ANS) (2018). Scientific Opinion on the Safety of Green Tea Catechins. EFSA J..

[40] Vieux F., Maillot M., Rehm C.D., Drewnowski A. (2020). Flavonoid Intakes in the US Diet are Linked to Higher Socioeconomic Status and to Tea Consumption: Analyses of NHANES 2011–2016 Data. J. Nutr..

[41] Health Canada Summary Safety Review—Green Tea Extract-Containing Natural Health Products—Assessing the Potential Risk of Liver Injury (Hepatotoxicity). https://www.canada.ca/en/health-canada/services/drugs-health-products/medeffect-canada/safety-reviews/green-tea-extract-containing-natural-health-products-assessing-potential-risk-liver-injury.html.

[42] Hu J., Webster D., Cao J., Shao A. (2018). The safety of green tea and green tea extract consumption in adults—Results of a systematic review. Regul. Toxicol. Pharm..

[43] Yates A.A., Erdman J.W., Shao A., Dolan L.C., Griffiths J.C. (2017). Bioactive nutrients—Time for tolerable upper intake levels to address safety. Regul. Toxicol. Pharm..

[44] Dekant W., Fujii K., Shibata E., Morita O., Shimotoyodome A. (2017). Safety assessment of green tea based beverages and dried green tea extracts as nutritional supplements. Toxicol. Lett..

[45] Oketch-Rabah H.A., Roe A.L., Rider C.V., Bonkovsky H.L., Giancaspro G.I., Navarro V., Paine M.F., Betz J.M., Marles R.J., Casper S. (2020). United States Pharmacopeia (USP) comprehensive review of the hepatotoxicity of green tea extracts. Toxicol. Rep..

[46] Matsuyama T., Tanaka Y., Kamimaki I., Nagao T., Tokimitsu I. (2008). Catechin safely improved higher levels of fatness, blood pressure, and cholesterol in children. Obes. Silver Spring.

[47] Huang J., Wang Y., Xie Z., Zhou Y., Zhang Y., Wan X. (2014). The anti-obesity effects of green tea in human intervention and basic molecular studies. Eur. J. Clin. Nutr..

[48] Chen I.J., Liu C.Y., Chiu J.P., Hsu C.H. (2016). Therapeutic effect of high-dose green tea extract on weight reduction: A randomized, double-blind, placebo-controlled clinical trial. Clin. Nutr..

[49] Xu X.Y., Zhao C.N., Li B.Y., Tang G.Y., Shang A., Gan R.Y., Feng Y.B., Li H.B. (2021). Effects and mechanisms of tea on obesity. Crit. Rev. Food Sci. Nutr..

[50] Asbaghi O., Fouladvand F., Gonzalez M.J., Aghamohammadi V., Choghakhori R., Abbasnezhad A. (2021). Effect of Green Tea on Anthropometric Indices and Body Composition in Patients with Type 2 Diabetes Mellitus: A Systematic Review and Meta-Analysis. Complement. Med. Res..

[51] Lin Y., Shi D., Su B., Wei J., Găman M.A., Sedanur Macit M., Borges do Nascimento I.J., Guimaraes N.S. (2020). The effect of green tea supplementation on obesity: A systematic review and dose-response meta-analysis of randomized controlled trials. Phytother. Res..

[52] Phung O.J., Baker W.L., Matthews L.J., Lanosa M., Thorne A., Coleman C.I. (2010). Effect of green tea catechins with or without caffeine on anthropometric measures: A systematic review and meta-analysis. Am. J. Clin. Nutr..

[53] Haghighatdoost F., Nobakht M.G.B.F., Hariri M. (2018). Effect of green tea on plasma leptin and ghrelin levels: A systematic review and meta-analysis of randomized controlled clinical trials. Nutrition.

[54] Haghighatdoost F., Nobakht M.G.B.F., Hariri M. (2017). Effect of Green Tea on Plasma Adiponectin Levels: A Systematic Review and Meta-analysis of Randomized Controlled Clinical Trials. J. Am. Coll. Nutr..

[55] Mansour-Ghanaei F., Hadi A., Pourmasoumi M., Joukar F., Golpour S., Najafgholizadeh A. (2018). Green tea as a safe alternative approach for nonalcoholic fatty liver treatment: A systematic review and meta-analysis of clinical trials. Phytother. Res..

[56] Zheng X.X., Xu Y.L., Li S.H., Hui R., Wu Y.J., Huang X.H. (2013). Effects of green tea catechins with or without caffeine on glycemic control in adults: A meta-analysis of randomized controlled trials. Am. J. Clin. Nutr..

[57] Liu K., Zhou R., Wang B., Chen K., Shi L.Y., Zhu J.D., Mi M.T. (2013). Effect of green tea on glucose control and insulin sensitivity: A meta-analysis of 17 randomized controlled trials. Am. J. Clin. Nutr..

[58] Wang X., Tian J., Jiang J., Li L., Ying X., Tian H., Nie M. (2014). Effects of green tea or green tea extract on insulin sensitivity and glycaemic control in populations at risk of type 2 diabetes mellitus: A systematic review and meta-analysis of randomised controlled trials. J. Hum. Nutr. Diet..

[59] Yu J., Song P., Perry R., Penfold C., Cooper A.R. (2017). The Effectiveness of Green Tea or Green Tea Extract on Insulin Resistance and Glycemic Control in Type 2 Diabetes Mellitus: A Meta-Analysis. Diabetes. Metab. J..

[60] Xu R., Bai Y., Yang K., Chen G. (2020). Effects of green tea consumption on glycemic control: A systematic review and meta-analysis of randomized controlled trials. Nutr. Metab..

[61] Asbaghi O., Fouladvand F., Gonzalez M.J., Ashtary-Larky D., Choghakhori R., Abbasnezhad A. (2021). Effect of green tea on glycemic control in patients with type 2 diabetes mellitus: A systematic review and meta-analysis. Diabetes. Metab. Syndr..

[62] Kim A., Chiu A., Barone M.K., Avino D., Wang F., Coleman C.I., Phung O.J. (2011). Green tea catechins decrease total and low-density lipoprotein cholesterol: A systematic review and meta-analysis. J. Am. Diet. Assoc..

[63] Onakpoya I., Spencer E., Heneghan C., Thompson M. (2014). The effect of green tea on blood pressure and lipid profile: A systematic review and meta-analysis of randomized clinical trials. Nutr. Metab. Cardiovasc. Dis..

[64] Xu R., Yang K., Li S., Dai M., Chen G. (2020). Effect of green tea consumption on blood lipids: A systematic review and meta-analysis of randomized controlled trials. Nutr. J..

[65] Yuan F., Dong H., Fang K., Gong J., Lu F. (2018). Effects of green tea on lipid metabolism in overweight or obese people: A meta-analysis of randomized controlled trials. Mol. Nutr. Food Res..

[66] Asbaghi O., Fouladvand F., Moradi S., Ashtary-Larky D., Choghakhori R., Abbasnezhad A. (2020). Effect of green tea extract on lipid profile in patients with type 2 diabetes mellitus: A systematic review and meta-analysis. Diabetes. Metab. Syndr..

[67] Li D., Wang R., Huang J., Cai Q., Yang C.S., Wan X., Xie Z. (2019). Effects and Mechanisms of Tea Regulating Blood Pressure: Evidences and Promises. Nutrients.

[68] Xu R., Yang K., Ding J., Chen G. (2020). Effect of green tea supplementation on blood pressure: A systematic review and meta-analysis of randomized controlled trials. Med. Baltim..

[69] Khalesi S., Sun J., Buys N., Jamshidi A., Nikbakht-Nasrabadi E., Khosravi-Boroujeni H. (2014). Green tea catechins and blood pressure: A systematic review and meta-analysis of randomised controlled trials. Eur. J. Nutr..

[70] Li G., Zhang Y., Thabane L., Mbuagbaw L., Liu A., Levine M.A., Holbrook A. (2015). Effect of green tea supplementation on blood pressure among overweight and obese adults: A systematic review and meta-analysis. J. Hypertens..

[71] Marcason W. (2013). What is green coffee extract?. J. Acad. Nutr. Diet..

[72] Lu H., Tian Z., Cui Y., Liu Z., Ma X. (2020). Chlorogenic acid: A comprehensive review of the dietary sources, processing effects, bioavailability, beneficial properties, mechanisms of action, and future directions. Compr. Rev. Food Sci. Food Saf..

[73] Naveed M., Hejazi V., Abbas M., Kamboh A.A., Khan G.J., Shumzaid M., Ahmad F., Babazadeh D., FangFang X., Modarresi-Ghazani F. (2018). Chlorogenic acid (CGA): A pharmacological review and call for further research. Biomed. Pharm..

[74] Farah A., de Paula Lima J. (2019). Consumption of Chlorogenic Acids through Coffee and Health Implications. Beverages.

[75] Olthof M.R., Hollman P.C., Buijsman M.N., van Amelsvoort J.M., Katan M.B. (2003). Chlorogenic acid, quercetin-3-rutinoside and black tea phenols are extensively metabolized in humans. J. Nutr..

[76] Integrated Laboratory Systems Chlorogenic Acid and Caffeine Acid: Review of Toxicological Literature. https://ntp.niehs.nih.gov/ntp/htdocs/chem_background/exsumpdf/chlorogenicacid_508.pdf.

[77] Soni & Associates Inc. Evaluation of the Generally Recognized as Safe (GRAS) Status of Coffeeberry (R) Coffee Fruit Extract as a Food Ingredient. https://www.fda.gov/media/135526/download.

[78] Asbaghi O., Sadeghian M., Nasiri M., Khodadost M., Shokri A., Panahande B., Pirouzi A., Sadeghi O. (2020). The effects of green coffee extract supplementation on glycemic indices and lipid profile in adults: A systematic review and dose-response meta-analysis of clinical trials. Nutr. J..

[79] Morvaridi M., Rayyani E., Jaafari M., Khiabani A., Rahimlou M. (2020). The effect of green coffee extract supplementation on cardio metabolic risk factors: A systematic review and meta-analysis of randomized controlled trials. J. Diabetes. Metab. Disord..

[80] Nikpayam O., Najafi M., Ghaffari S., Jafarabadi M.A., Sohrab G., Roshanravan N. (2019). Effects of green coffee extract on fasting blood glucose, insulin concentration and homeostatic model assessment of insulin resistance (HOMA-IR): A systematic review and meta-analysis of interventional studies. Diabetol. Metab. Syndr..

[81] Roshan H., Nikpayam O., Sedaghat M., Sohrab G. (2018). Effects of green coffee extract supplementation on anthropometric indices, glycaemic control, blood pressure, lipid profile, insulin resistance and appetite in patients with the metabolic syndrome: A randomised clinical trial. Br. J. Nutr..

[82] Blum J., Lemaire B., Lafay S. (2007). Effect of a green decaffeinated coffee extract on glycaemia. A pilot prospective clinical study. Nutrafoods.

[83] Mansour A., Mohajeri-Tehrani M.R., Samadi M., Qorbani M., Merat S., Adibi H., Poustchi H., Hekmatdoost A. (2021). Effects of supplementation with main coffee components including caffeine and/or chlorogenic acid on hepatic, metabolic, and inflammatory indices in patients with non-alcoholic fatty liver disease and type 2 diabetes: A randomized, double-blind, placebo-controlled, clinical trial. Nutr. J..

[84] Asbaghi O., Sadeghian M., Rahmani S., Mardani M., Khodadost M., Maleki V., Pirouzi A., Talebi S., Sadeghi O. (2020). The effect of green coffee extract supplementation on anthropometric measures in adults: A comprehensive systematic review and dose-response meta-analysis of randomized clinical trials. Complement. Med..

[85] Chen Y., Zhao Y., Wang Y., Nazary-Vannani A., Clark C.C.T., Sedanur Macit M., Khani V., Zhang Y. (2020). The influence of green coffee bean extract supplementation on blood glucose levels: A systematic review and dose-response meta-analysis of randomized controlled trials. Phytother. Res..

[86] Han B., Nazary-Vannani A., Talaei S., Clark C.C.T., Rahmani J., Rasekhmagham R., Kord-Varkaneh H. (2019). The effect of green coffee extract supplementation on blood pressure: A systematic review and meta-analysis of randomized controlled trials. Phytother. Res..

[87] Bosso H., Barbalho S.M., de Alvares Goulart R., Otoboni A. (2021). Green coffee: Economic relevance and a systematic review of the effects on human health. Crit. Rev. Food Sci. Nutr..

[88] Arias N., Arboleya S., Allison J., Kaliszewska A., Higarza S.G., Gueimonde M., Arias J.L. (2020). The Relationship between Choline Bioavailability from Diet, Intestinal Microbiota Composition, and Its Modulation of Human Diseases. Nutrients.

[89] Wiedeman A.M., Barr S.I., Green T.J., Xu Z., Innis S.M., Kitts D.D. (2018). Dietary Choline Intake: Current State of Knowledge Across the Life Cycle. Nutrients.

[90] Institute of Medicine (1998). Dietary Reference Intakes for Thiamin, Riboflavin, Niacin, Vitamin B6, Folate, Vitamin B12, Pantothenic Acid, Biotin, and Choline.

[91] EFSA NDA Panel (2016). EFSA Panel on Dietetic Products, Nutrition and Allergies. Scientific Opinion on Dietary Reference Values for Choline. EFSA J..

[92] Amazon Best Sellers in Choline Vitamin Supplements. https://www.amazon.com/gp/bestsellers/hpc/6939007011.

[93] Elsawy G., Abdelrahman O., Hamza A. (2014). Effect of choline supplementation on rapid weight loss and biochemical variables among female taekwondo and judo athletes. J. Hum. Kinet..

[94] Killgore W.D., Ross A.J., Kamiya T., Kawada Y., Renshaw P.F., Yurgelun-Todd D.A. (2010). Citicoline affects appetite and cortico-limbic responses to images of high-calorie foods. Int. J. Eat. Disord..

[95] Gao X., Wang Y., Randell E., Pedram P., Yi Y., Gulliver W., Sun G. (2016). Higher Dietary Choline and Betaine Intakes Are Associated with Better Body Composition in the Adult Population of Newfoundland, Canada. PLoS ONE.

[96] Jafari A., Jalilpiran Y., Suitor K., Bellissimo N., Azadbakht L. (2021). The association of dietary choline and betaine and anthropometric measurements among Iranian children: A cross-sectional study. BMC Pediatr..

[97] Leermakers E.T., Moreira E.M., Kiefte-de Jong J.C., Darweesh S.K., Visser T., Voortman T., Bautista P.K., Chowdhury R., Gorman D., Bramer W.M. (2015). Effects of choline on health across the life course: A systematic review. Nutr. Rev..

[98] Nakamura T., Nakamura S., Karoji N., Aikawa T., Suzuki O. (1967). Hepatic function tests in heavy drinkers among workmen. Tohoku. J. Exp. Med..

[99] Buchman A.L., Ament M.E., Sohel M., Dubin M., Jenden D.J., Roch M., Pownall H., Farley W., Awal M., Ahn C. (2001). Choline deficiency causes reversible hepatic abnormalities in patients receiving parenteral nutrition: Proof of a human choline requirement: A placebo-controlled trial. JPEN J. Parenter. Enter. Nutr..

[100] Guerrerio A.L., Colvin R.M., Schwartz A.K., Molleston J.P., Murray K.F., Diehl A., Mohan P., Schwimmer J.B., Lavine J.E., Torbenson M.S. (2012). Choline intake in a large cohort of patients with nonalcoholic fatty liver disease. Am. J. Clin. Nutr..

[101] Yu D., Shu X.O., Xiang Y.B., Li H., Yang G., Gao Y.T., Zheng W., Zhang X. (2014). Higher dietary choline intake is associated with lower risk of nonalcoholic fatty liver in normal-weight Chinese women. J. Nutr..

[102] Corbin K.D., Zeisel S.H. (2012). Choline metabolism provides novel insights into nonalcoholic fatty liver disease and its progression. Curr. Opin. Gastroenterol..

[103] Sherriff J.L., O’Sullivan T.A., Properzi C., Oddo J.L., Adams L.A. (2016). Choline, Its Potential Role in Nonalcoholic Fatty Liver Disease, and the Case for Human and Bacterial Genes. Adv. Nutr..

[104] Naghipour S., Cox A.J., Peart J.N., Du Toit E.F., Headrick J.P. (2021). Trimethylamine N-oxide: Heart of the microbiota-CVD nexus?. Nutr. Res. Rev..

[105] Lemaitre R.N., Jensen P.N., Wang Z., Fretts A.M., McKnight B., Nemet I., Biggs M.L., Sotoodehnia N., de Oliveira Otto M.C., Psaty B.M. (2021). Association of Trimethylamine N-Oxide and Related Metabolites in Plasma and Incident Type 2 Diabetes: The Cardiovascular Health Study. JAMA Netw. Open.

[106] Rashvand S., Mobasseri M., Tarighat-Esfanjani A. (2020). Effects of Choline and Magnesium Concurrent Supplementation on Coagulation and Lipid Profile in Patients with Type 2 Diabetes Mellitus: A Pilot Clinical Trial. Biol. Trace Elem. Res..

[107] Wallace J.M.W., McCormack J.M., McNulty H., Walsh P.M., Robson P.J., Bonham M.P., Duffy M.E., Ward M., Molloy A.M., Scott J.M. (2012). Choline supplementation and measures of choline and betaine status: A randomised, controlled trial in postmenopausal women. Br. J. Nutr..

[108] Olthof M.R., van Vliet T., Verhoef P., Zock P.L., Katan M.B. (2005). Effect of homocysteine-lowering nutrients on blood lipids: Results from four randomised, placebo-controlled studies in healthy humans. PLoS Med..

[109] Devaraj R.D., Reddy C.K., Xu B. (2019). Health-promoting effects of konjac glucomannan and its practical applications: A critical review. Int. J. Biol. Macromol..

[110] Tester R.F., Al-Ghazzewi F.H. (2016). Beneficial health characteristics of native and hydrolysed konjac (Amorphophallus konjac) glucomannan. J. Sci. Food Agric..

[111] FDA FDA Grants Citizen Petition on Glucomannan as a Dietary Fiber. https://www.fda.gov/food/cfsan-constituent-updates/fda-grants-citizen-petition-glucomannan-dietary-fiber.

[112] Hornick B., Birkett A., Liska D.J. (2013). The Fiber Deficit, Part 3—Beyond Traditional Fiber Sources. Nutr. Today.

[113] Mortensen A., Aguilar F., Crebelli R., Di Domenico A., Frutos M.J., Galtier P., Gott D., Gundert-Remy U., Lambre C., EFSA Panel on Food Additives Nutrient Sources added to Food (ANS) (2017). Re-evaluation of konjac gum (E 425 i) and konjac glucomannan (E 425 ii) as food additives. EFSA J..

[114] Onakpoya I., Posadzki P., Ernst E. (2014). The efficacy of glucomannan supplementation in overweight and obesity: A systematic review and meta-analysis of randomized clinical trials. J. Am. Coll. Nutr..

[115] Zalewski B.M., Chmielewska A., Szajewska H. (2015). The effect of glucomannan on body weight in overweight or obese children and adults: A systematic review of randomized controlled trials. Nutrition.

[116] Martino F., Martino E., Morrone F., Carnevali E., Forcone R., Niglio T. (2005). Effect of dietary supplementation with glucomannan on plasma total cholesterol and low density lipoprotein cholesterol in hypercholesterolemic children. Nutr. Metab. Cardiovasc. Dis..

[117] Vido L., Facchin P., Antonello I., Gobber D., Rigon F. (1993). Childhood obesity treatment: Double blinded trial on dietary fibres (glucomannan) versus placebo. Padiatr. Padol..

[118] Zalewski B.M., Szajewska H. (2019). No Effect of Glucomannan on Body Weight Reduction in Children and Adolescents with Overweight and Obesity: A Randomized Controlled Trial. J. Pediatr..

[119] Henry D.A., Mitchell A.S., Aylward J., Fung M.T., McEwen J., Rohan A. (1986). Glucomannan and risk of oesophageal obstruction. Br. Med. J. Clin. Res. Ed..

[120] FDA 21 CFR 201.319. Water-Soluble Gums, Hydrophilic Gums, and Hydrophilic Mucilloids (Including, but not Limited to Agar, Alginic Acid, Calcium Polycarbophil, Caeboxymethylcellulose Sodium, Carrageenan, Chondrus, Glucomannas ((B-1,4 Linked) Polymannose Acetate), Guar Gum, Karaya Gum, Kelp, Methylcellulose, Plantago Seed (Psyllium), Polycarbophil Tragacanth, and Xanthan Gum) as Active Ingredients; Requried Warnings and Directions. https://www.ecfr.gov/current/title-21/chapter-I/subchapter-C/part-201/subpart-G/section-201.319.

[121] EFSA Panel on Dietetic Products, Nutrition and Allergies (NDA) (2010). Scientific Opinion on the substantiation of health claims related to konjac mannan (glucomannan) and reduction of body weight (ID 854, 1556, 3725), reduction of post-prandial glycaemic responses (ID 1559), maintenance of normal blood glucose concentrations (ID 835, 3724), maintenance of normal (fasting) blood concentrations of triglycerides (ID 3217), maintenance of normal blood cholesterol concentrations (ID 3100, 3217), maintenance of normal bowel function (ID 834, 1557, 3901) and decreasing potentially pathogenic gastro-intestinal microorganisms (ID 1558) pursuant to Article 13(1) of Regulation (EC) No 1924/2006. EFSA J..

[122] Bessell E., Maunder A., Lauche R., Adams J., Sainsbury A., Fuller N.R. (2021). Efficacy of dietary supplements containing isolated organic compounds for weight loss: A systematic review and meta-analysis of randomised placebo-controlled trials. Int. J. Obes..

[123] Calatayud M., Van den Abbeele P., Ghyselinck J., Marzorati M., Rohs E., Birkett A. (2021). Comparative Effect of 22 Dietary Sources of Fiber on Gut Microbiota of Healthy Humans in vitro. Front. Nutr..

[124] Li Y., Kang Y., Du Y., Chen M., Guo L., Huang X., Li T., Chen S., Yang F., Yu F. (2022). Effects of Konjaku Flour on the Gut Microbiota of Obese Patients. Front. Cell Infect. Microbiol..

[125] Kaats G.R., Bagchi D., Preuss H.G. (2015). Konjac Glucomannan Dietary Supplementation Causes Significant Fat Loss in Compliant Overweight Adults. J. Am. Coll. Nutr..

[126] Behera S.S., Ray R.C. (2016). Konjac glucomannan, a promising polysaccharide of Amorphophallus konjac K. Koch in health care. Int. J. Biol. Macromol..

[127] Sood N., Baker W.L., Coleman C.I. (2008). Effect of glucomannan on plasma lipid and glucose concentrations, body weight, and blood pressure: Systematic review and meta-analysis. Am. J. Clin. Nutr..

[128] Ho H.V.T., Jovanovski E., Zurbau A., Blanco Mejia S., Sievenpiper J.L., Au-Yeung F., Jenkins A.L., Duvnjak L., Leiter L., Vuksan V. (2017). A systematic review and meta-analysis of randomized controlled trials of the effect of konjac glucomannan, a viscous soluble fiber, on LDL cholesterol and the new lipid targets non-HDL cholesterol and apolipoprotein B. Am. J. Clin. Nutr..

[129] Ueno H., Haraguchi N., Azuma M., Shiiya T., Noda T., Ebihara E., Uehira Y., Uchida T., Sasaba K., Nakamura M. (2021). Active Consumption of Konjac and Konjac Products Improves Blood Glucose Control in Patients with Type 2 Diabetes Mellitus. J. Am. Coll. Nutr..

[130] Yoshida A., Kimura T., Tsunekawa K., Araki O., Ushiki K., Ishigaki H., Shoho Y., Suda I., Hiramoto S., Murakami M. (2020). Glucomannan Inhibits Rice Gruel-Induced Increases in Plasma Glucose and Insulin Levels. Ann. Nutr. Metab..

[131] Basith S., Cui M., Hong S., Choi S. (2016). Harnessing the Therapeutic Potential of Capsaicin and Its Analogues in Pain and Other Diseases. Molecules.

[132] Huang X.F., Xue J.Y., Jiang A.Q., Zhu H.L. (2013). Capsaicin and its analogues: Structure-activity relationship study. Curr. Med. Chem..

[133] Torres-Ugalde Y.C., Romero-Palencia A., Román-Gutiérrez A.D., Ojeda-Ramírez D., Guzmán-Saldaña R.M.E. (2020). Caffeine Consumption in Children: Innocuous or Deleterious? A Systematic Review. Int. J. Environ. Res. Public Health.

[134] Whiting S., Derbyshire E., Tiwari B.K. (2012). Capsaicinoids and capsinoids. A potential role for weight management? A systematic review of the evidence. Appetite.

[135] Kwon Y. (2021). Estimation of Dietary Capsaicinoid Exposure in Korea and Assessment of Its Health Effects. Nutrients.

[136] Lopez-Carrillo L., Lopez-Cervantes M., Robles-Diaz G., Ramirez-Espitia A., Mohar-Betancourt A., Meneses-Garcia A., Lopez-Vidal Y., Blair A. (2003). Capsaicin consumption, Helicobacter pylori positivity and gastric cancer in Mexico. Int. J. Cancer.

[137] EFSA Panel on Dietetic Products, Nutrition and Allergies (NDA) (2012). Scientific Opinion on Dihydrocapsiate. EFSA J..

[138] FDA NDI 739, Dihydrocapsiate from Ajinomoto North America. https://www.regulations.gov/document/FDA-2012-S-1178-0020.

[139] EFSA Panel on Dietetic Products, Nutrition and Allergies (NDA) (2019). Safety of phenylcapsaicin as a novel food pursuant to Regulation (EU) 2015/2283. EFSA J..

[140] Ludy M.J., Moore G.E., Mattes R.D. (2012). The effects of capsaicin and capsiate on energy balance: Critical review and meta-analyses of studies in humans. Chem. Senses.

[141] Shin K.O., Moritani T. (2007). Alterations of autonomic nervous activity and energy metabolism by capsaicin ingestion during aerobic exercise in healthy men. J. Nutr. Sci. Vitam..

[142] Shin K.O., Moritani T. (2008). Capsaicin supplementation fails to modulate autonomic and cardiac electrophysiologic activity during exercise in the obese: With variants of UCP2 and UCP3 polymorphism. J. Sports Sci. Med..

[143] Lejeune M.P., Kovacs E.M., Westerterp-Plantenga M.S. (2003). Effect of capsaicin on substrate oxidation and weight maintenance after modest body-weight loss in human subjects. Br. J. Nutr..

[144] Jang H.H., Lee J., Lee S.H., Lee Y.M. (2020). Effects of Capsicum annuum supplementation on the components of metabolic syndrome: A systematic review and meta-analysis. Sci. Rep..

[145] Deshpande J., Jeyakodi S., Juturu V. (2016). Tolerability of Capsaicinoids from Capsicum Extract in a Beadlet Form: A Pilot Study. J. Toxicol..

[146] Pabalan N., Jarjanazi H., Ozcelik H. (2014). The impact of capsaicin intake on risk of developing gastric cancers: A meta-analysis. J. Gastrointest. Cancer.

[147] Luo L., Yan J., Wang X., Sun Z. (2021). The correlation between chili pepper consumption and gastric cancer risk: A meta-analysis. Asia Pac. J. Clin. Nutr..

[148] Du Y., Lv Y., Zha W., Hong X., Luo Q. (2021). Chili Consumption and Risk of Gastric Cancer: A Meta-Analysis. Nutr. Cancer.

[149] Shi Z., Riley M., Taylor A.W., Page A. (2017). Chilli consumption and the incidence of overweight and obesity in a Chinese adult population. Int. J. Obes. Lond..

[150] EFSA Panel on Dietetic Products, Nutrition and Allergies (NDA) (2011). Scientific Opinion on the substantiation of heatlh claims related to capsaicin and maintenance of body weight after loss (ID 2039, 2041, 2042), increase in carbohydrate oxidation (ID 2040), and contribution to normal hair growth (ID 2044) pursuant to Article 13 (1) of Regulation (EC) No. 1924/2006. EFSA J..

[151] Zsiborás C., Mátics R., Hegyi P., Balaskó M., Pétervári E., Szabó I., Sarlós P., Mikó A., Tenk J., Rostás I. (2018). Capsaicin and capsiate could be appropriate agents for treatment of obesity: A meta-analysis of human studies. Crit. Rev. Food Sci. Nutr..

[152] Fuse S., Endo T., Tanaka R., Kuroiwa M., Ando A., Kume A., Yamamoto A., Kuribayashi K., Somekawa S., Takeshita M. (2020). Effects of Capsinoid Intake on Brown Adipose Tissue Vascular Density and Resting Energy Expenditure in Healthy, Middle-Aged Adults: A Randomized, Double-Blind, Placebo-Controlled Study. Nutrients.

[153] Nirengi S., Homma T., Inoue N., Sato H., Yoneshiro T., Matsushita M., Kameya T., Sugie H., Tsuzaki K., Saito M. (2016). Assessment of human brown adipose tissue density during daily ingestion of thermogenic capsinoids using near-infrared time-resolved spectroscopy. J. Biomed. Opt..

[154] Whiting S., Derbyshire E.J., Tiwari B. (2014). Could capsaicinoids help to support weight management? A systematic review and meta-analysis of energy intake data. Appetite.

[155] van Avesaat M., Troost F.J., Westerterp-Plantenga M.S., Helyes Z., Le Roux C.W., Dekker J., Masclee A.A., Keszthelyi D. (2016). Capsaicin-induced satiety is associated with gastrointestinal distress but not with the release of satiety hormones. Am. J. Clin. Nutr..

[156] Liang W., Lan Y., Chen C., Song M., Xiao J., Huang Q., Cao Y., Ho C.T., Lu M. (2021). Modulating effects of capsaicin on glucose homeostasis and the underlying mechanism. Crit. Rev. Food. Sci. Nutr..

[157] Qin Y., Ran L., Wang J., Yu L., Lang H.D., Wang X.L., Mi M.T., Zhu J.D. (2017). Capsaicin Supplementation Improved Risk Factors of Coronary Heart Disease in Individuals with Low HDL-C Levels. Nutrients.

[158] Urbina S.L., Roberts M.D., Kephart W.C., Villa K.B., Santos E.N., Olivencia A.M., Bennett H.M., Lara M.D., Foster C.A., Purpura M. (2017). Effects of twelve weeks of capsaicinoid supplementation on body composition, appetite and self-reported caloric intake in overweight individuals. Appetite.

[159] Shirani F., Foshati S., Tavassoly M., Clark C.C.T., Rouhani M.H. (2021). The effect of red pepper/capsaicin on blood pressure and heart rate: A systematic review and meta-analysis of clinical trials. Phytother. Res..

[160] Amini M.R., Sheikhhossein F., Bazshahi E., Hajiaqaei M., Shafie A., Shahinfar H., Azizi N., Eghbaljoo Gharehgheshlaghi H., Naghshi S., Fathipour R.B. (2021). The effects of capsinoids and fermented red pepper paste supplementation on blood pressure: A systematic review and meta-analysis of randomized controlled trials. Clin. Nutr..

[161] Daniells S. CRN Survey: 80% of Americans are Now Using Dietary Supplements. https://www.nutraingredients-usa.com/Article/2021/10/22/CRN-survey-80-of-Americans-are-now-using-dietary-supplements#.

